# Acute hepatic failure due to dengue: A case report

**DOI:** 10.1186/1757-1626-1-204

**Published:** 2008-10-02

**Authors:** Subhash Giri, Mukul P Agarwal, Vishal Sharma, Ankur Singh

**Affiliations:** 1 Department of Medicine, University College of Medical Sciences, Delhi, India

## Abstract

Dengue is an arboviral disease endemic in many parts of the world. Although it is known to cause hepatic involvement commonly, it only occasionally results in acute hepatic failure. We present the case of a young male who developed acute hepatic failure due to dengue. The differentials and the management is discussed.

## Background

Dengue is a common arboviral illness endemic in India. Hepatitis in patients of dengue is not uncommon. However dengue is only rarely considered as a cause of acute liver failure. We report a case of 22 year old male who developed deranged liver functions, coagulopathy and encephalopathy due to dengue.

## Case report

A 22 year old male, resident of Delhi presented to us in the month of September with three day history of high grade fever associated with chills and rigors, severe headache, myalgias, nausea and vomiting. There was no history of bleeding from any site. There was no history of alcohol intake or abuse in past and he was not exposed to any hepatotoxic drugs. There was no other significant past medical or surgical history. At the time of presentation the patient was febrile (40°C) and had subconjunctival hemorrhages in both eyes 1. Systemic examination was essentially normal. Investigations revealed a Hb of 11.5 gm%, TLC-6450/mm^3 ^and thrombocytopenia (platelet count-32000/cu mm). Chest roentgenogram and ECG were normal. Ultrasonography of abdomen revealed gall bladder edema and mild ascites.

**Figure 1:**
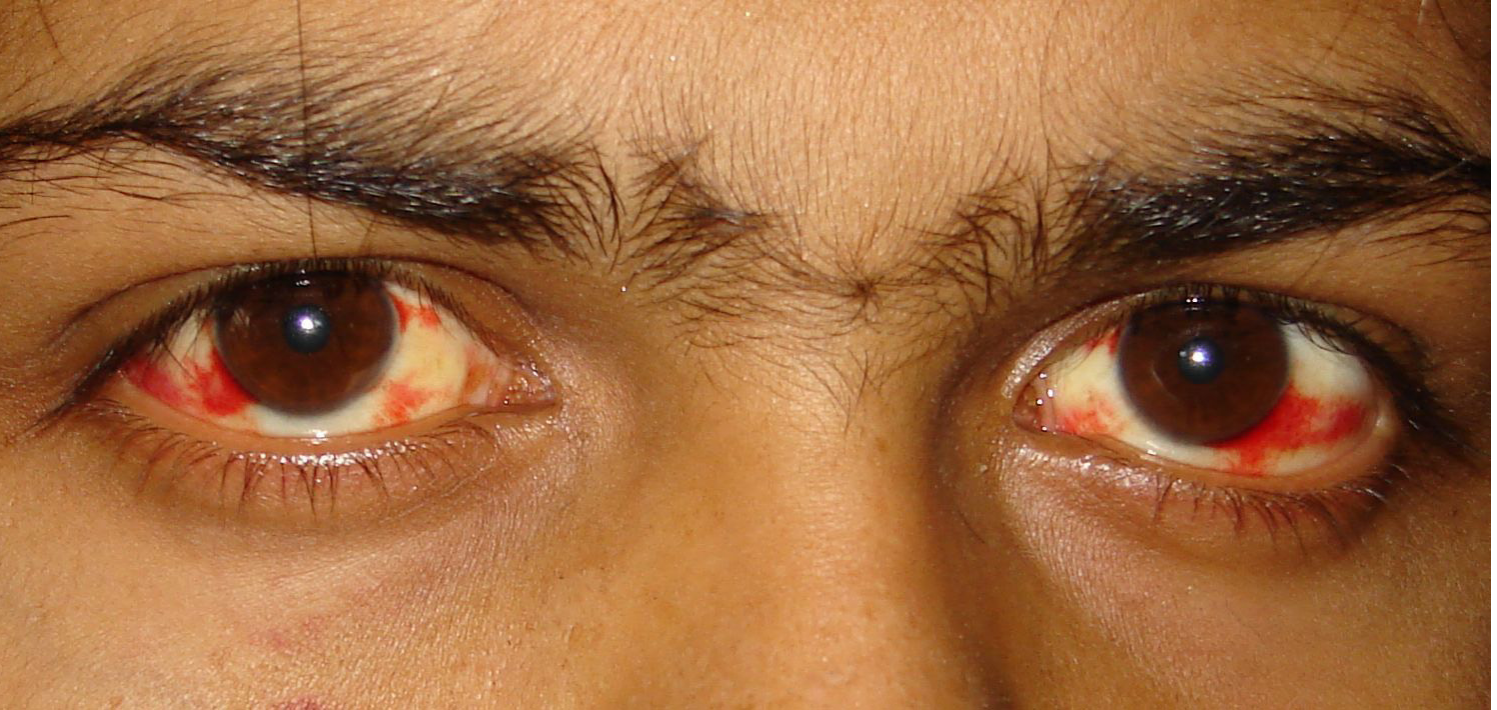
Subconjunctival haemorrhages in the patient with Dengue.

Two days later the patient developed hematuria but maintained a normal urine output. He became increasingly irritable and within a day developed disorientation to time, place and person. He had no neck rigidity or focal neurological deficit. The patient was anicteric but his liver dullness was reduced. Spleen was not palpable. His investigations at this stage revealed thrombocytopenia (platelet-18000/cu mm.) and coagulopathy (PT-22 seconds against control of 13 seconds), deranged liver functions (Serum Albumin-2.6, Total bilirubin-2.8, direct bilirubin-1.0, SGPT-868, SGOT-910, Alkaline Phosphatase-304). His renal functions and electrolytes were normal.

In view of deranged liver functions, evidence of coagulopathy and altered sensorium a possibility of acute liver failure was kept. Tests for viral serologies including IgM Anti HAV, HBsAg, IgM Anti HBc, Anti HCV and IgM Anti HEV were negative. Blood cultures revealed no growth. Serology for Leptospirosis and Widal test were negative. Peripheral smears for malaria and pLDH antigen for Plasmodium vivax and Plasmodium falciparum were negative. In view of endemicity of dengue in Delhi, thrombocytopenia, gall bladder edema and hepatitis a diagnosis of dengue was suspected and a IgM dengue serology was obtained which was positive. The patient was managed with platelets and fresh frozen plasma transfusions and his sensorium improved. Liver functions, platelets and coagulation profile gradually improved and the patient was discharged.

## Discussion

Dengue is a viral illness characterized by high grade fever, hemorrhagic manifestations and features of shock. However, in recent times the reports of rare manifestations of dengue have become more common and may include central nervous system manifestations (encephalopathy.), liver and renal failure [1]. Liver enzyme elevations are common in dengue. Usually the SGOT levels are more than SGPT levels probably due to skeletal muscle injury. Occasionally jaundice is also seen [2]. Our patient had a low serum albumin (2.6 gm %), however this has been seen in an earlier study involving patients with dengue who had evidence of hepatitis [3].

Few reports of acute liver failure have come from across the globe. A study in pediatric population found that dengue was the most common cause of acute liver failure in Thailand [4]. Even from India reports indict dengue as a common cause of acute hepatic failure in pediatric age group especially during periods of epidemic [5]. However the scenario in adult population is not as well established and dengue is not usually considered as a differential in patients of acute liver failure [6].

The diagnosis of acute liver failure due to dengue should be considered in patients with evidence of acute onset alteration in sensorium and with feature suggestive of dengue (high grade fever, hemorrhagic manifestations, thrombocytopenia, plasma leak syndrome characterized by increasing hematocrit, gall bladder wall edema etc.). The differentials to be considered include acute viral hepatitis, malaria, leptospirosis, drug reactions (Table 1). In our patient the presence of a febrile illness, thrombocytopenia, liver enzyme elevations, and ascites were all consistent with possibility of dengue. The management of acute liver failure in dengue is primarily supportive (see Table 1).

**Table 1:** Differrentials of acute hepatic failure due to dengue

	VIRAL	MALARIA	LEPTOSPIROSIS	DRUG	DENGUE
HIGH GRADE FEVER	-	+	+	-	+
HEMATOCRIT	N	FALLS	N	N	RISES
SGPT	+++	+	+	+++	+++
ARF	-	+	+	-	-
PLASMA LEAK	-	RARE	-	-	++
PLATELET	N	FALLS	N	N	FALLS

Therefore our patient had evidence of dengue infection and developed acute liver failure during the course of his disease. Hence dengue should be considered as a possible cause of acute liver failure in endemic areas if other viral markers are negative.

## Competing interests

The authors declare that they have no competing interests.

## Authors' contributions

SG and MPA were involved in conception and designing, revision of manuscript and final approval of manuscript. VS and AS were involved in data acquisition and manuscript writing, revision and final approval of the manuscript.

## Consent

Written informed consent was obtained from the patient for publication of this case report and accompanying images. A copy of the written consent is available for review by the Editor-in-Chief of this journal.
